# From guidelines to patient-centred action

**DOI:** 10.1007/s12471-025-01971-2

**Published:** 2025-07-18

**Authors:** Pim van der Harst

**Affiliations:** Department of Cardiology, Division of Heart and Lungs, University Medical Centre Utrecht, Utrecht, The Netherlands

The *Netherlands Heart Journal* recently began publishing NVVC endorsements of new ESC guidelines, reflecting our shared ambition to support, high-quality cardiovascular care in the Netherlands. As previously noted [1], this initiative helps translate international evidence into local applications. In this issue, we present another strong example. Martens et al. clearly endorse the 2023 ESC guideline on cardiovascular disease with diabetes [2]. Their roadmap links SCORE2-Diabetes risk assessment, initiation of SGLT2 inhibitors and GLP‑1 receptor agonists, and the updated Dutch CVRM guideline. They urge us to screen proactively for dysglycaemia, kidney dysfunction and early heart failure, recognising risk is the first step toward reducing it.

This issue also presents three studies offering new clinical insights across the care spectrum.

Balder et al. report their experience with Impella CP cardiogenic shock patients [3]. Outcomes were promising in those considered for durable LVAD. However, when escalation was ruled out due to comorbidities, mortality reached 100%. The message is clear: timing, selection, and structured multidisciplinary decision-making are key to meaningful haemodynamic support.

Vloet et al. examine physical activity patterns in over 1,000 patients with cardiovascular disease [4]. While exercise levels remained stable, sedentary time increased significantly and remained elevated post-pandemic. This challenges us to address sitting time more explicitly in rehabilitation and chronic care.

Finally, Bragança et al. show that segmental multifrequency bioelectrical impedance analysis (BIA) reveals subclinical congestion in over half of apparently stable HFrEF patients [5]. These BIA-derived measures strongly predicted deterioration and may offer a simple, scalable tool in outpatient follow-up.

This issue brings what we need most: clear guidance when possible and real-world lessons where practice still evolves.
